# Laser-Scribing Optimization for Sprayed SnO_2_-Based Perovskite Solar Modules on Flexible Plastic Substrates

**DOI:** 10.1021/acsaem.1c00140

**Published:** 2021-05-05

**Authors:** Babak Taheri, Francesca De Rossi, Giulia Lucarelli, Luigi Angelo Castriotta, Aldo Di Carlo, Thomas M. Brown, Francesca Brunetti

**Affiliations:** † CHOSE, Department of Electronic Engineering, Università degli Studi di Roma Tor Vergata, Via del Politecnico 1, Rome 00133, Italy; ‡ LASE−Laboratory for Advanced Solar Energy, National University of Science and Technology MISiS, Moscow 119049, Russia; § Institute for Structure of Matter, National Research Council (CNR-ISM), via del Fosso del Cavaliere 100, Rome 00133, Italy

**Keywords:** flexible perovskite solar cells, p1-p2-p3 laser scribing, perovskite module, large-area deposition, automized spray-coating, SnO_2_ electron transport layer, pet/ito substrate

## Abstract

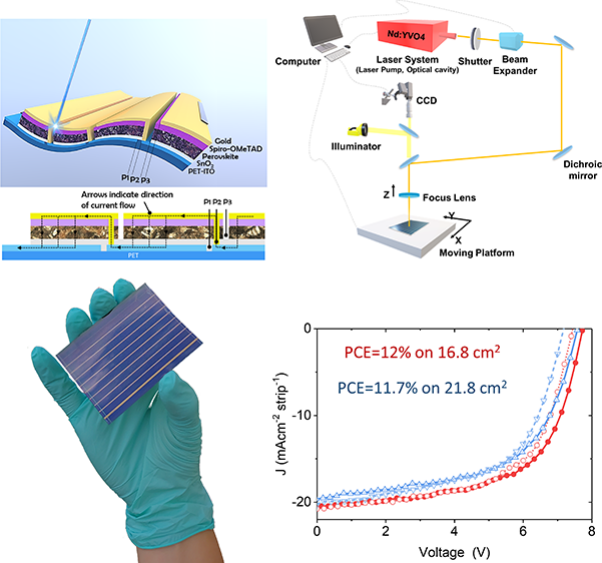

Flexible perovskite solar cells (FPSCs) are prime candidates for applications requiring a highly efficient, low-cost, lightweight, thin, and even foldable power source. Despite record efficiencies of lab-scale flexible devices (19.5% on a 0.1 cm^2^ area), scalability represents a critical factor toward commercialization of FPSCs. Large-area automized deposition techniques and efficient laser scribing procedures are required to enable a high-throughput production of flexible perovskite modules (FPSMs), with the latter being much more challenging compared to glass substrates. In this work, we introduce the combined concept of laser scribing optimization and automatized spray-coating of SnO_2_ layers. Based on a systematic variation of the incident laser power and a comprehensive morphological and electrical analysis of laser-based cell interconnections, optimal scribing parameters are identified. Furthermore, spray-coating is used to deposit uniform compact SnO_2_ films on large-area (>120 cm^2^) plastic substrates. FPSCs with spray-coated SnO_2_ show comparable performance as spin-coated cells, delivering up to 15.3% efficiency on small areas under 1 sun illumination. When upscaling to large areas, FPSMs deliver 12% power conversion efficiency (PCE) and negligible hysteresis on 16.8 cm^2^ and 11.7% PCE on a 21.8 cm^2^ active area. Our perovskite devices preserved 78% efficiency when the active area increased from 0.1 to 16.8 cm^2^, demonstrating that our combined approach is an effective strategy for large-area manufacturing of perovskite devices on flexible substrates.

## 1 Introduction

Among all the conventional and new-generation photovoltaic technologies, organic–inorganic metal halide perovskite solar cells (PSCs) exhibit appealing benefits that include high efficiency of up to 25.5%,^1^ low-temperature fabrication, and solution processability,^2−5^ which makes this technology compatible with most flexible substrates.^6^ Lightweight, bendable perovskite solar devices can be used in various applications, like transportable electronic chargers, flexible displays, biomedical devices, conformable sensors, and wearable electronic textiles, therefore attracting significant attention both from the scientific and industrial community.

Currently, flexible perovskite solar cells (FPSCs) have reached over 19% power conversion efficiency (PCE) on small area,^7,8^ adopting the planar structure as the preferred architecture because of its simplicity and low-temperature fabrication.^9,10^ Most of the studies have been carried out on small cells with an active area of approximately 0.1 cm^2^,^11−13^ while fewer studies are available regarding large-area FPSCs.^14,15^ In particular, since 2018, only few studies have been reported regarding flexible perovskite solar modules (FPSMs,^16−19^ see Table 1) realized on plastic substrates, with a PCE of 8.8% on a 12 cm^2^ active area^16^ and 14.89% on a 16 cm^2^ active area.^17^ When the solar cell or module area increases, the efficiency inevitably decreases. The loss of efficiency arises from a combination of several factors, including higher series resistance of the contacts, in particular the transparent conductive oxide (TCO), non-uniform coating over a large area, recombination pathways, and shunting losses due to non-optimal interconnections.^20,21^

**Table 1:** Recent Progress in Flexible Perovskite Solar Modules on Plastic Substrates, in which Interconnections Were Obtained by Means of a Laser Scribing Process^a^

ETL coating method	device structure	series interconnection	active area (cm^2^)	*J*_SC_ (mA/cm^2^)	*V*_OC_ (V)	FF (%)	PCE (%)	ref (year)
ALD	PET/ITO/TiO2/meso-TiO2/perovskite/spiro-OMeTAD/Au	masking/laser	7.92	1.3	3.39	71	3.1	(22)(2015)
spin	PEN/ITO/MFGO/perovskite/ PC61BM/BCP/Ag	chemical etching/masking	10	4.34	3.76	49.1	8.1	(19)(2016)
spin	PET/ITO/SnO_2_/meso-TiO_2_/perovskite/spiro-OMeTAD/Au	laser	12	3.12	5.014	55.9	8.8	(16)(2018)
spin	PET/ITO/SnO_2_/perovskite/spiro-OMeTAD/Au	laser	10	6.479	3.075	62	12.31	(18)(2018)
slot-die	PET/ITO/SnO_2_/perovskite/spiro-OMeTAD/Au	laser	16.09	3.28	6.727	69	14.89	(17)(2018)
spray	PET/ITO/SnO_2_/perovskite/spiro-OMeTAD/Au	laser	16.84	2.55	7.72	60.8	12.0	this work
spray	PET/ITO/SnO_2_/perovskite/spiro-OMeTAD/Au	laser	21.84	2.45	7.57	57.7	11.7	this work

a ALD = atomic layer deposition, PET = polyethylene terephthalate, ITO = indium tin oxide, SnO_2_ = tin oxide, PEN = polyethylene naphthalate, MFGO = fluorinated reduced graphene oxide, PC61BM = [6,6]-phenyl-C_61_-butyric acid methyl ester, and BCP = bathocuproine.

**Figure 1:**
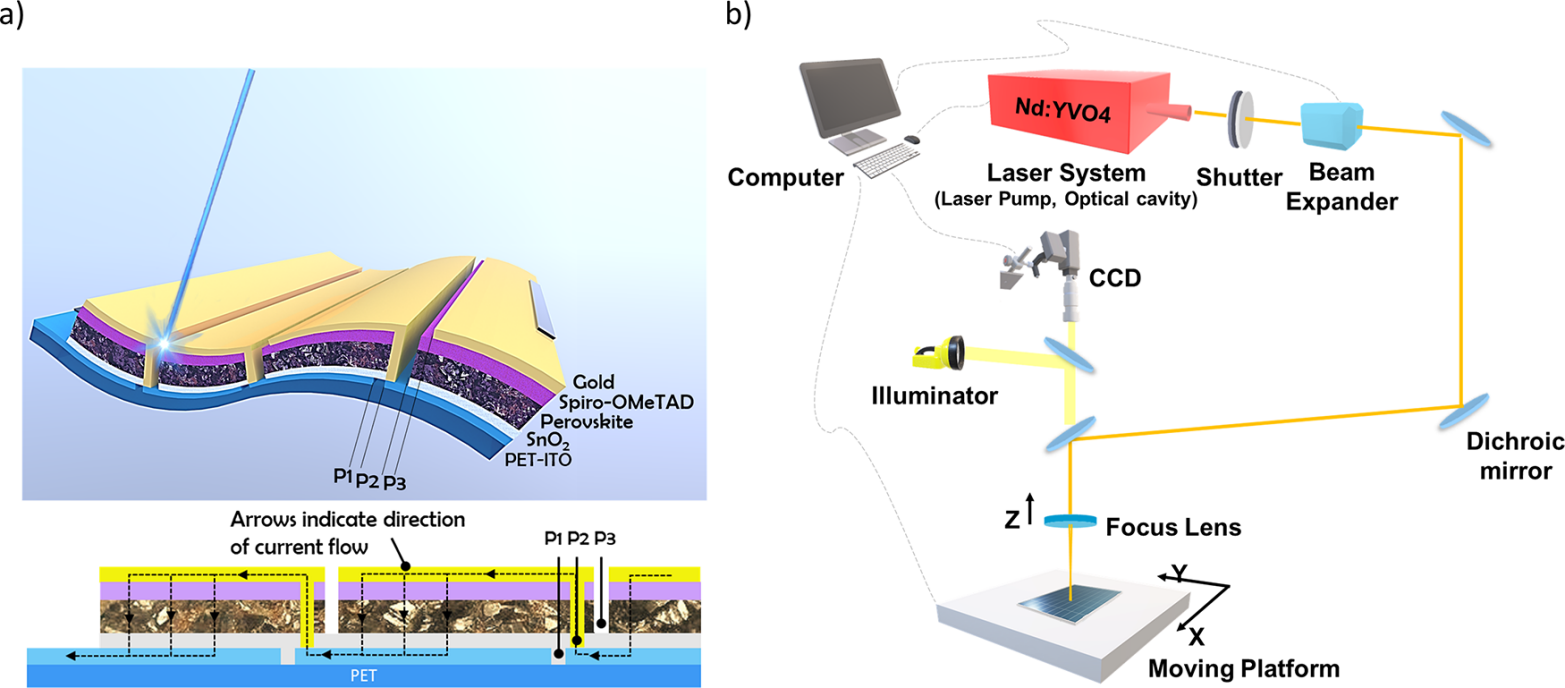
(a) Schematic sketch of the interconnection structure of a flexible perovskite module, highlighting P1, P2, and P3 laser scribes. (b) Diagram of the laser scribing setup used in this study for perovskite solar devices.

The lack of reports on perovskite modules on flexible, plastic substrates can be attributed to the additional challenge, besides the quality control of the active layers on large areas, of obtaining optimal interconnections between cells on flexible substrates via laser processing, a much more difficult task compared to rigid glass substrates.^23^ The series interconnection of adjacent cells in monolithic modules is normally achieved by alternating layer deposition and laser patterning steps (Figure 1). First, to define the width of the individual cells, parallel scribes are obtained by removing the transparent conductive oxide (TCO) constituting the bottom electrode (P1 laser patterning step). After the deposition of the perovskite absorber and electron and hole transport layers (ETLs and HTLs), the entire stack is selectively removed (second scribing step, P2) beside the P1 scribe line to expose the TCO. Finally, after the deposition of the counter electrode, this layer is ablated (third laser scribing step, P3) next to the P2 scribe to electrically separate the sub-cells of the module.

Recently, intense research activity has been devoted to the investigation and optimization of laser processing for perovskite solar modules on rigid glass substrate, with considerable progress achieved since the first pioneering work by Matteocci et al.^24^ and Wang et al.^25^ evaluated the scribing performance of a picosecond laser of 532 nm wavelength for mesoscopic perovskite modules based on a compact-TiO_2_ ETL and fluorine-doped tin oxide (FTO) as a bottom contact. They elucidated the predominant mechanisms of the scribing process, such as layer-by-layer ablation and lift-off mechanism, depending on the laser illumination direction. Regarding planar perovskite solar modules, Schultz et al.^26^ identified the optimal process parameters for successful laser-processed series interconnection by a systematic variation of the incident laser fluence and a comprehensive electrical, morphological, and compositional analysis of the P2 scribed area. Kosasih et al.^27^ clarified the interaction between P3 laser pulses and the performance of perovskite solar modules, highlighting the need to minimize material damage by carefully tuning both laser parameters and device fabrication procedures. Bayer et al. in two different works^28,29^ studied the mechanism of thin-film removal and the resulting surface modifications, such as film smoothening and decomposition of the MAPbI_3_, by using nanosecond (ns), picosecond (ps), and femtosecond (fs) laser pulses in order to obtain a complete removal of the perovskite film without damaging the FTO bottom contact. Turan et al.^30^ investigated methods to characterize and optimize the P2 process for the removal of the perovskite absorber, by means of nanosecond-pulsed ultraviolet (UV), green, and infrared (IR) laser ablation. They showed that process optimization based on a morphological evaluation only is not sufficient to ensure good interconnections and high solar module performance.

Although many groups have addressed the laser ablation on rigid glass substrates, there is currently no study available regarding the optimization of this process on flexible substrates. Material ablation on plastic films differs greatly from that on rigid glass, as a result of the difference in light reflection and in robustness of the plastic films under the illumination of high energy photons. Therefore, the optimization of laser processes on rough or curved plastic substrates is a great challenge and of crucial importance for the development of large-area, low-cost, and flexible perovskite modules.

A previous work on flexible modules has shown the feasibility of using different types of lasers to create series interconnections in monolithic structures of perovskite solar modules on plastic substrates. For example, Di Giacomo et al.^22^ used a CO_2_ laser for the fabrication of the first flexible perovskite photovoltaic module, while Dagar et al.^16^ and Bu et al.^17^ showed the use of green and IR lasers to make module interconnections.

Here, a nanosecond raster scanning UV laser with a 355 nm of wavelength was used for the P1, P2, and P3 processes. We characterized morphologically and electrically the scribe area of perovskite solar modules on a flexible PET/ITO substrate and optimized the laser patterning of FPSM by means of optical microscopy, scanning electron microscopy (SEM), and series resistance analysis.

Moreover, with the aim of developing a scalable fabrication process for the module, we investigated the spray-coating technique as an alternative large-area deposition method for the deposition of SnO_2_ nanoparticles (SnO_2_-NP) compact films. SnO_2_ has come to the fore as an excellent ETL candidate in highly efficient PSCs due to its transparency to the visible light, higher electron mobility than TiO_2_,^32^ photostability and compatibility with the perovskite absorbers,^10,33^ as well as low-temperature processing. Up to now, some research groups have tried to deposit SnO_2_ with large-area techniques such as atomic-layer deposition (ALD),^34^ chemical bath deposition (CBD)^35^ on a conducive glass substrate, and slot-die coating^17^ on flexible PET/ITO. Nevertheless, spin-coating is used in most cases as the preferred deposition technique.^36^ For planar n-i-p PSCs, an aqueous suspension of SnO_2_ nanoparticles is usually spin-coated followed by an annealing step at low temperatures (≤150 °C).^7^ However, spin-coating is not a scalable technique. Spray-coating, on the contrary, is a low-cost manufacturing process, compatible for industrial scale and high-throughput production, which has been already demonstrated to be effective for depositing thin layers of SnO_2_ on glass, leading to a PCE as high as 17%.^37^ In addition to transport layers, for which spray-coating has been demonstrated as a highly effective approach,^38^ considerable progress has been made also for spray-coating of the perovskite layer, enabling the fabrication of fully spray-coated cells.^39^ Highly reproducible coatings have been obtained by means of this technique, showing very small variations in performance across batches due to the high uniformity and easy control of the deposition parameters.^40,41^ Expected developments to further improve the quality of spray-coated films should tackle the two main limitations of this technique, i.e., the requirements for low concentrated precursor solutions and the fairly limited operation window for the crystallization of the active layer by the combined tuning of ink properties (i.e., concentration, viscosity, solvents,) and processing parameters (e.g., substrate temperature, which affects the wettability and the evaporation of the solvent) or by using advanced two-step spray methods.^38^

In this paper, we report for the first time the use of this technique on large PET/ITO substrates, i.e., 10 × 12.5 cm^2^, compared to standard spin-coated ones. We found that sprayed SnO_2_ as the ETL can be a worthy replacement for conventional spin-coated films, reducing device hysteresis, and leading to a PCE of 15.3% on a flexible substrate and an active area of 0.1 cm^2^. By combining the optimized laser process and the large-area spray deposition of SnO_2_, we obtained flexible modules with 12% PCE and negligible hysteresis on 16.8 cm^2^ and 11.7% PCE on 21.8 cm^2^ of active area. Moreover, with this combined optimization, we gained a 36% efficiency improvement compared to our previous work^23^ for a 40% larger active area.

## 2 Results

### 2.1 Optimization of the P2 Laser Scribing Process

For flexible perovskite solar cells, different structures are reported in the literature.^15,42^ In this work, the following n-i-p planar architecture is considered: PET/ITO/SnO_2_/Cs_0.05_FA_0.80_MA_0.15_Pb(I_0.85_Br_0.15_)_3_/spiro-OMeTAD/Au. Our investigation focuses on the P2 laser scribing step, that is the removal of the whole heterogeneous stack on the top of the ITO electrode, prior to the Au evaporation, as schematically represented in Figure 1. The main parameter to take into account when we talk about laser scribing is fluence:^43^ this parameter is defined as the ratio between single pulse energy and spot area. Single pulse energy is defined as the ratio between the average power and repetition rate. The spot area of the laser pulse is defined by the laser scanning speed and the raster scanning distance (RSD), a parameter that defines the distance between two adjacent scribe lines. In our work, to vary the fluence, we varied both power and RSD while keeping the repetition rate constant and equal to 80 kHz.

#### 2.1.1 Morphology and Topography of P2 Single Scribe Lines

The multilayer to be removed consisted of 40 nm of SnO_2_ compact layer, ∼600 nm of perovskite film, and ∼250 nm of spiro-OMeTAD layer. Thickness values were measured from FIB-SEM cross-sectional images (see Figure S1).

**Figure 2:**
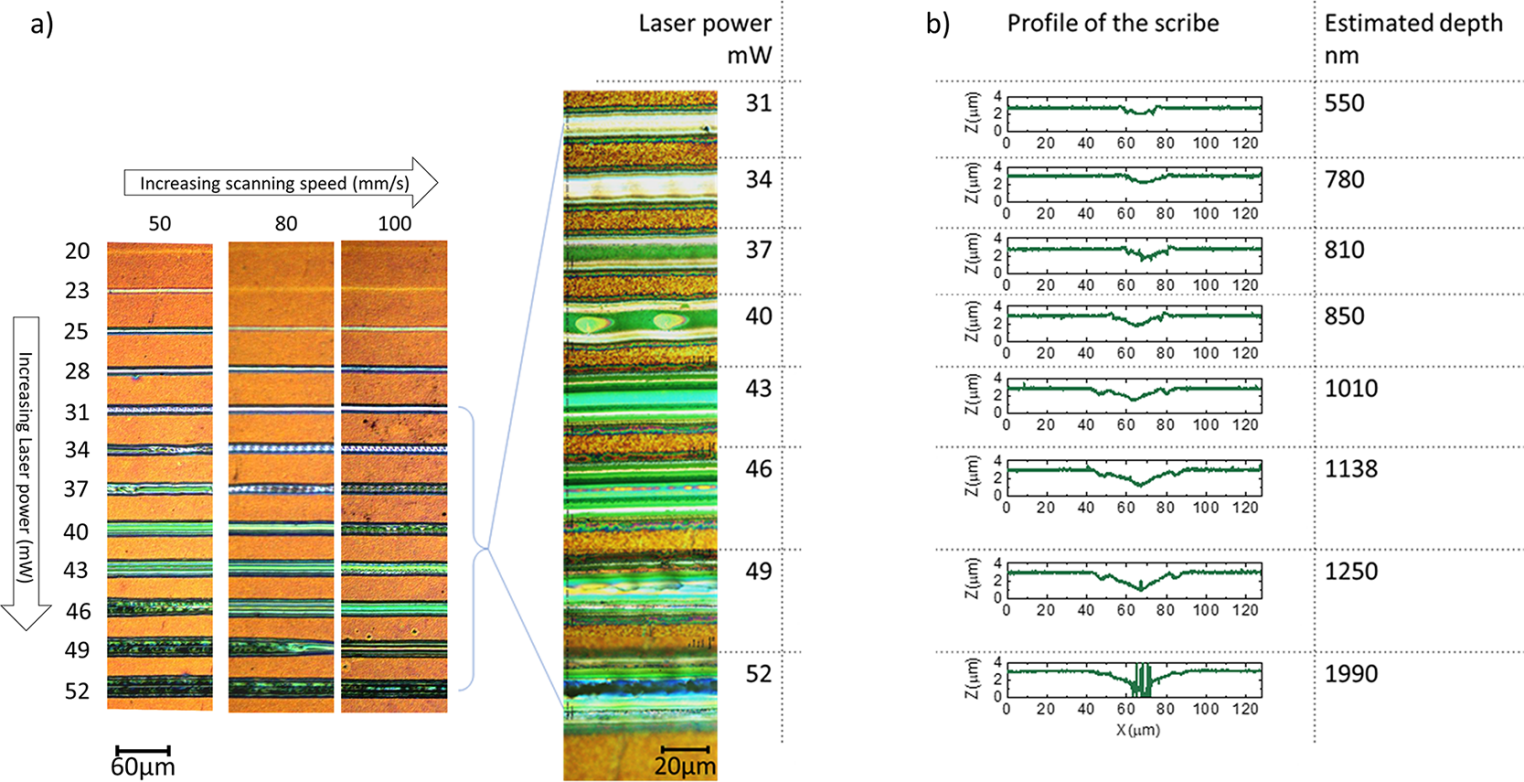
(a) Optical microscope images of P2 lines, laser scribed on PET/ITO/SnO_2_/Cs_0.05_FA_0.80_MA_0.15_Pb(I_0.85_Br_0.15_)_3_/spiro-OMeTAD, at different scanning speeds and increasing laser power; the appearance of the green color implies the SnO_2_ ITO has been exposed, removing the layers above. (b) Laser scanning confocal microscopy profiles of laser-patterned P2 scribed lines at laser impulse power ranging between 31 and 52 mW, to evaluate the effectiveness of the ∼1 μm-thick stack removal.

At first, single scribe lines obtained by different values of laser pulse power and laser scanning speed were evaluated; Figure 2a shows optical microscopy images and profiles of such laser-patterned P2 scribe lines on PET/ITO/SnO_2_/perovskite/spiro-OMeTAD samples. The depth of each scribe, that is the thickness of the film removed, was estimated by a non-destroying method employing 3D laser scanning confocal microscopy (LSCM). The upper surface of the stack showed visible variation already after scribing at the lowest laser pulse power, i.e., 20 mW first scribe line at the top of Figure 2a, regardless of the scanning speed. The removal of each layer, due to the ablation process, is due to the decomposition of the materials, which is probably related to photothermal processes^44^ that have a specific power threshold for each material considered. The comparison of different scanning speeds, namely, 50, 80, and 100 mm/s (Figure 2a), reveals that the highest speed tested, i.e., 100 mm/s, allows a finer tuning of the selective material ablation, in particular in the 31 to 52 mW power range, as it pushes to higher power levels the undesired damage of the ITO (darker, wide lines on the bottom of Figure 2a). Interestingly, the laser at 355 nm can selectively remove the spiro-OMeTAD layer at low laser power levels, as observed in some lines in Figure 2a at 20–25 mW. By increasing the laser pulse fluence, surface modification is enhanced, and the scribe width increases, also bringing the PET substrate to a higher exposition, as shown in Figure 2a. Due to the optical transparency of the HTM, the laser beam will be mainly absorbed by the perovskite after passing through the HTM layer, thus leading to a rapid phase change and ablation of the perovskite material. At a high power laser pulse (>46 mW), the risk of damaging the bottom ITO contact is higher, whereas low powers (<23 mW) are not sufficient to remove even the first layer, i.e., the spiro-OMeTAD film. After identifying the optimal ablation scanning speed, which was set at 100 mm/s, a narrower range of laser pulse fluences was investigated in order to analyze the difference in surface modification. The optical images and micro-profiles of a laser ablated active layer, processed at 100 mm/s speed, show that, by tuning the laser power from 34 to 46 mW, the surface modifies its appearance, and the amount of removed material increases with laser power, as expected. At 37 mW laser power, the depth of the micro-profile is around 810 nm, implying that both spiro-OMeTAD and perovskite have been removed and that the dark green color of the scribe corresponds to the exposed underlying SnO_2_ layer. It is fair to assume that the removal of a part of SnO_2_ is driven by the rapid expansion of the overlying perovskite layer, but there is minimal laser power to overcome the cohesion between SnO_2_ nanoparticles. On the other hand, laser scribes with a pulse power from 43 to 46 mW remove effectively the whole spiro/perovskite/SnO_2_ stack, estimated to be ∼900 nm by the cross-sectional SEM image reported in Figure S1. In fact, the depth of the scribe is about 1.010–1.140 μm (see Figure 2b), implying the complete removal of all HTL, perovskite, and ETL but also some damage of the small portion of ITO underneath (∼100 nm deep and localized at the center of the scribe line), hit by the peak of the Gaussian shaped laser beam.

Minor damage was initiated in the ITO layer at the laser power of 49 mW, while at 52 mW, serious damage of the ITO film was observed, as clearly showed by the microscopy image of Figure 2a. Laser power above 46 mW was therefore considered not suitable for P2 scribing while we focused on the range of 43 to 46 mW for the rest of the work.

#### 2.1.2 Raster Scanning Distance (RSD) Optimization of P2 Laser-Scribed Strips

To ensure a good electrical interconnection between the top electrode of a cell and the bottom electrode of the adjacent cell, one single-laser scribe line is not enough,^45,46^ whereas creating a strip of contiguous overlapping single lines is potentially more effective. Such a process of etching single lines one beside the other is controlled by the raster scanning distance (RSD), a parameter that defines the distance between one scribe line and another. It must be noted that, even if a single line scribe does not damage the bottom electrode, overlapping scribe lines may cause it due to incubation effects.^47^ Alternatively, spaced lines may not create the necessary area for top connection and consequently induce high recombination at the interconnection interfaces, therefore causing high series resistance and low FF in solar modules. An adequate combination of laser pulse power and RSD can ablate the irradiated material before heat transfer occurs, avoiding damage to the bottom contact at the interconnection region.^46,48^ Thus, it is crucial to optimize the RSD parameter of P2 ablation in order to decrease the modules performance losses.

**Figure 3:**
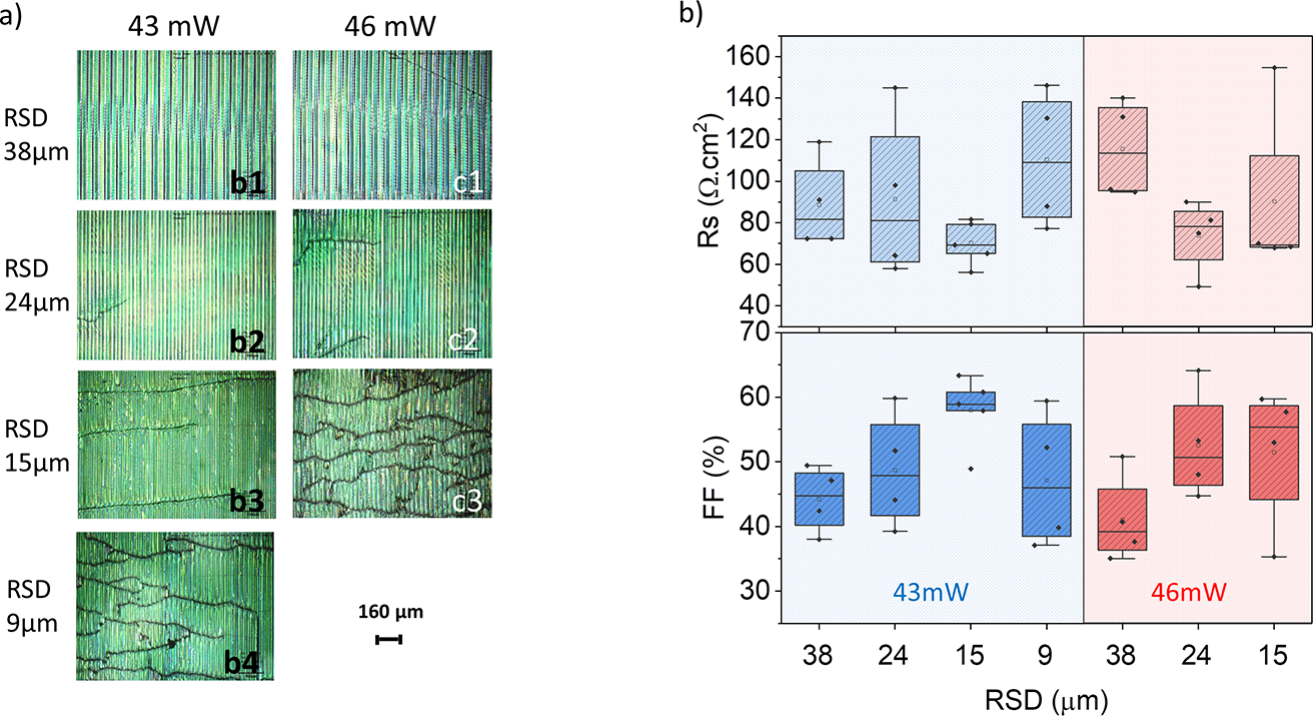
(a) Microscopy images of P2 laser-scribed strip processed with parameters chosen from Figure 2 and varying the raster scanning distance (RSD). (b) Series resistance and FF obtained from the *J*–*V* curves of mini modules, consisting of three cells interconnected in series on a 2.5 × 2.5 cm^2^ substrate, resulting in 2.5 cm^2^ active area, fabricated with selected laser parameters.

Laser pulses at 43 or 46 mW power and raster scanning speed of 100 mm/s were used to laser scribe rectangular areas of 1 mm × 2 mm in order to evaluate the effectiveness of the P2 step while tuning the RSD. Figure 3a shows the optical images of the scribed strips: by decreasing RSD, regardless of the power, the risk of heat transfer increases, causing visible cracks in the ITO. Unlike glass substrates, plastic substrates are much more sensitive to temperature; thus, it is mandatory to increase the RSD when increasing laser pulse power to avoid damaging the ITO.

The effectiveness of the P2 scribing parameters was evaluated by the electrical characterization of mini modules, made of 3 cells interconnected in series on a 2.5 × 2.5 cm^2^ substrate, resulting in 2.457 cm^2^ active area (Figure S2), fabricated by varying the RSD. The P2 scribing process was performed at 43 and 46 mW of power, tuning the RSD from 9 to 50 μm. The P3 laser patterning is related to the top-electrode insulation.

The FF and series resistance of the devices, which are sensitive to the quality of the interconnections and therefore provide a good indication of the quality of the RSD parameter, were obtained from the *J*–*V* measurements of 28 flexible mini modules and are shown in Figure 3b. The series resistance was determined from the inverse of the slope of the *J*–*V* curve at the intersection with the *x*-axis; a low value of series resistance is considered as a good indication of a successful P2 patterning.

For both 43 and 46 mW, at higher RSD such as 38 μm, the performance of the mini modules is rather low due to high series resistance, which causes poor FF. Decreasing the RSD reduces series resistance and increases the FF. Further decreasing of the RSD (i.e., 9 μm) causes again a higher series resistance accompanied by a lower FF, very likely due to cracks in the ITO electrode, visible under the microscope (Figure 3a).

By using an RSD of 15 μm and a laser pulse power of 43 mW (labeled as b3 in Figure 3a), the best results were achieved, with the highest FF of 64% and lowest series resistance of 49 Ω·cm^2^, as shown in Figure 3b. These values highlight that the small portion of ITO removed, once it is covered with evaporated gold, that has a very low resistivity, i.e., 2.4·10^–8^ Ω·m, does not cause any particular contact issue since the current is still able to flow with the reduced ITO thickness, as shown in Figure 1a. The ratio between the P2 scribe width (300 micron) and ITO thickness does prevent any additional series resistance occurring in this process. The optimized P2 scribe generated good interconnection without introducing the significant degradation of the bottom electrode; the corresponding laser parameters (43 mW of laser power and 15 μm of RSD) were selected and adopted for the fabrication of larger modules.

### 2.2 Toward Large-Area Flexible Perovskite Modules

#### 2.2.1 Spray Deposition of SnO_2_ Layers on PET/ITO

**Figure 4:**
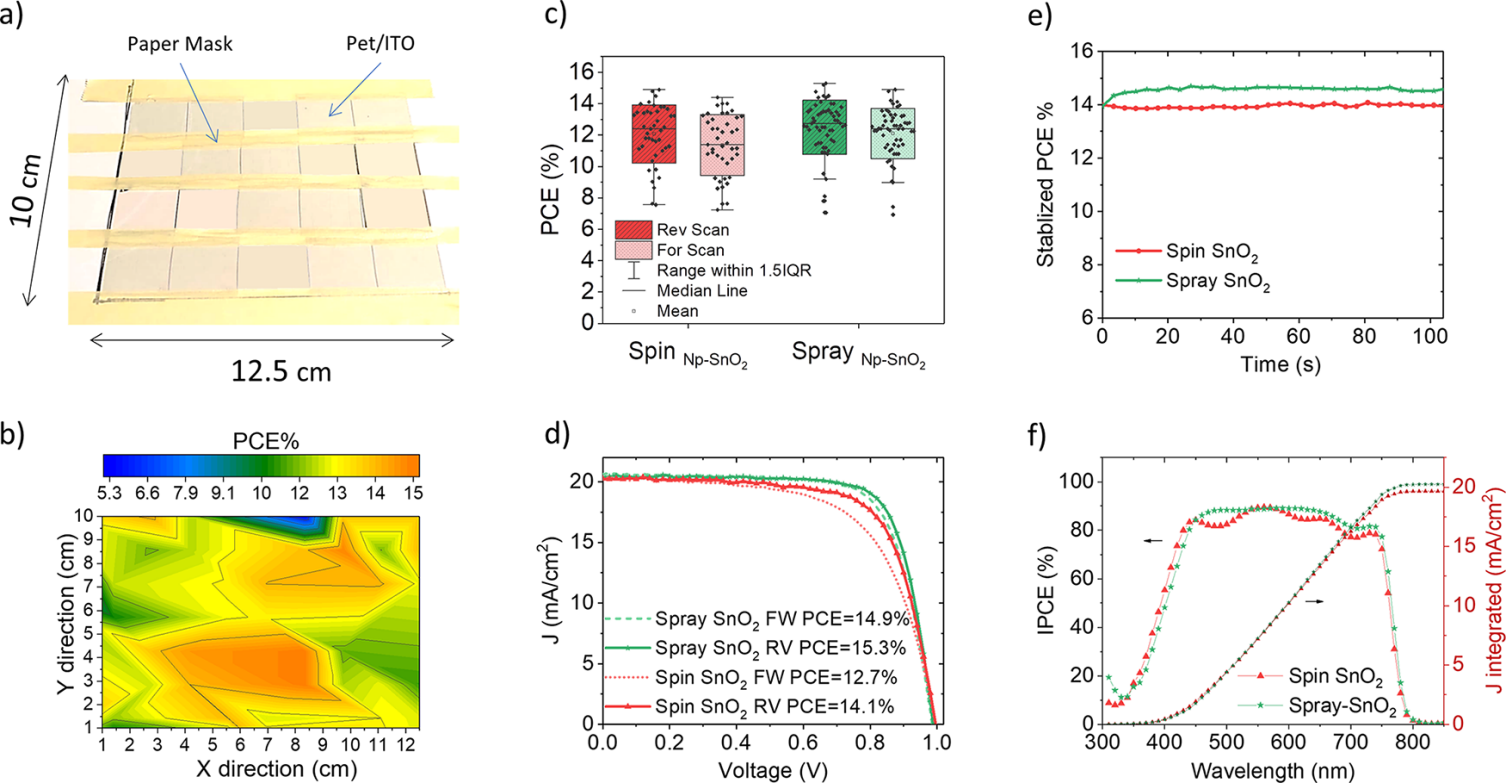
(a) Photograph of the flexible substrate on an automated spray deposition vacuum plate after the SnO_2_ film coating. (b) PCE map of spray SnO_2_-based FPSCs on a 10 × 12.5 cm^2^ flexible PET/ITO substrate. (c) Statistical results of PCE of spin-coated and spray-coated SnO_2_ based FPSCs. (d) Forward/reverse scan *J*–*V* curve of best FPSCs and (e) stabilized PCE at MPP based on spin-coated and spray-coated SnO_2_ measured under 1 sun 1.5 AM illumination. (f) IPCE spectra and integrated *J*_SC_ of spin-coated and spray-coated SnO_2_-based FPSCs.

To scale-up from mini modules to larger modules, automated spray-coating of water-based SnO_2_-NP dispersions was employed to deposit a compact ETL on a 10 × 12.5 cm^2^ PET/ITO substrate (Figure 4a), which was then divided into 20 smaller substrates (2.5 × 2.5 cm^2^ each). Optimized deposition parameter settings were used^37^ (see Section 4 for details).

To assess the uniformity of the sprayed SnO_2_ layer on the larger area, the transmittance spectra of the smaller substrates were recorded; a variation as low as 1.6% in average transmittance in the 350–850 nm range confirmed the uniformity of the deposition on large areas (see Figure S3).

Then, cells were fabricated on the 20 small-area spray-coated samples by spin-coating the perovskite and spiro-OMeTAD layers and by evaporating the gold top electrode. By knowing the initial position of each small substrate during the SnO_2_ deposition and by assigning to each location the PCE value of the correspondent device, a 2D map of the PCE was plotted: PCE spans from 7.4 to 15.3%, with just 2% standard deviation, as depicted in Figure 4b.

Reference cells with a standard spin-coated SnO_2_ ETL were also fabricated and compared to the cells with spray-coated SnO_2_ layers. The statistics of the PCE of the cells reported in Figure 4c show a slightly smaller deviation for spray-coated devices (1.72% relative variation from the average value compared to 1.85% of spin-coated cells). The average PCE of spray SnO_2_-based devices was 12.18%, which is comparable to that of spin-coated SnO_2_-based FPSCs (PCE 12.2%). *J*–*V* curves of the best spray and spin SnO_2_-based cells are reported in Figure 4d. The champion cell had a spray-coated SnO_2_ ETL and exhibited 15.3% PCE with 989 mV of *V*_OC_, 20.39 mA/cm^2^ of *J*_SC_, and 76% of FF. The best spin-coated SnO_2_ cell showed 14.8% PCE with 1013 mV of *V*_OC_, 19.48 mA/cm^2^ of *J*_SC_, and 74% of FF.

Spray-coated and spin-coated SnO_2_-based cells exhibited 14.6 and 13.9% stabilized PCE, respectively, which was measured by maximum power point tracking (MPPT, Figure 4e).

The analysis of the reverse and forward *J*–*V* scan and of the stabilized PCE shows that the hysteresis of FPSCs is reduced upon the introduction of a spray-coated SnO_2_ ETL; in particular, the hysteresis index, calculated as HI = (PCE_rev_ – PCE_for_)/PCE_rev_^49^ was reduced by 35% in relative terms, as shown in Figure S4a). Hysteresis behavior in perovskite solar devices might be due to slow transient capacitive current, dynamic trapping and de-trapping processes of charge carriers, and ion migration or ferroelectric polarization inside the device,^50−53^ which is common in a planar structure.^54,55^

**Figure 5:**
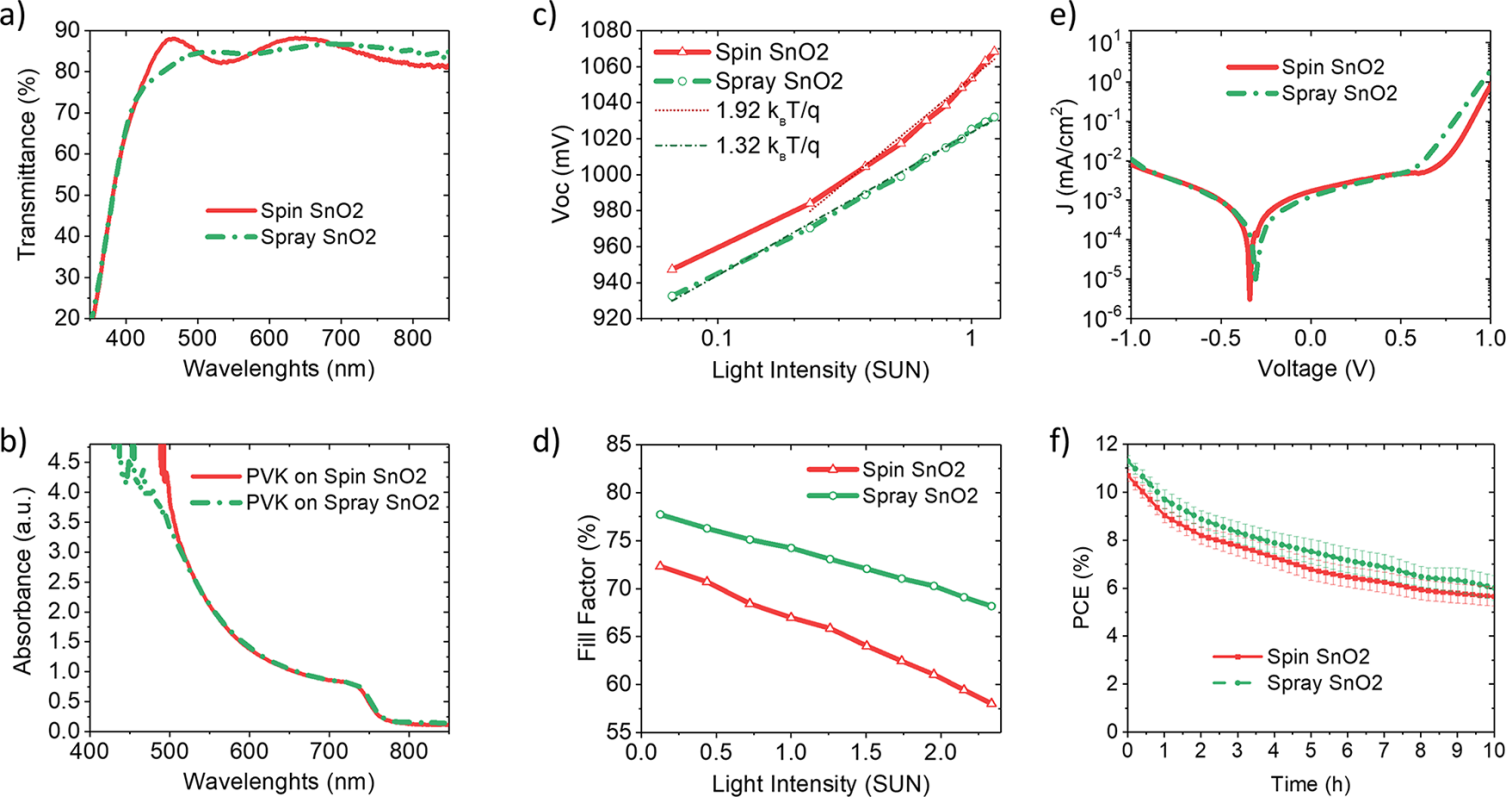
(a) Transmittance of SnO_2_ films on PET/ITO and (b) absorbance of perovskite layers grown on the SnO_2_ ETL. The (c) *V*_OC_ (light intensity) and (d) FF (light intensity) characteristics of spin and spray SnO_2_-based FPSCs. (e) *J*–*V* curves in the dark. (f) Light soaking stability of unsealed FPSCs based on spin-coated and spray-coated SnO_2_: maximum power point tracking under 1 sun illumination in air at 55 °C.

*J*_SC_ of both spray-coated and spin-coated SnO_2_-based cells calculated under 1 sun illumination (Figure S4b) was consistent with the integrated *J*_SC_ calculated from the incident photon-to-electron conversion efficiency (IPCE), as shown in Figure 4f. The IPCE of PSCs with a spray-coated SnO_2_ ETL is lower than that of spin-coated SnO_2_ cells in the low visible wavelength window (350–450 nm), but surpasses the latter in the orange to red wavelengths (600–800 nm) where the most important contributions are the light harvesting from the perovskite and the transport of the charges toward the contacts. This improvement could be either related to a better charge transport in the spray cells or can also be due to optical interference effects.^56^ Transparency of the spray-coated SnO_2_ film in the red region is slightly higher than that of spin-coated SnO_2_, as shown in Figure 5a. Nevertheless, there is no significant variation in terms of perovskite absorption, as confirmed by the absorption spectra of perovskite films grown on different SnO_2_ ETLs (Figure 5b). This means that the superior IPCE of spray-coated SnO_2_ based cells is probably due to the enhanced charge transport from the perovskite absorber to the ITO contact.

One of the phenomena that can suppress the hysteresis of perovskite cells is the reduction of capacitive effects due to the dynamic charge trapping/de-trapping process.^50^ As regards to the better electron transport in the cell with spray-coated SnO_2_, reduction in hysteresis might be due to a decrease in the trap states in SnO_2_ and at the SnO_2_/perovskite interface.

To further prove the effect of the electron transport and injection efficiency at the perovskite/SnO_2_ interface, we evaluated the electrical parameters of spin-coated and spray-coated SnO_2_-based cells vs illumination intensity, in addition to the *J*–*V* in the dark. Notably, spray-coated SnO_2_-based devices showed a better performance at low intensity light, which suggests that these cells might be interesting for indoor applications.^57^ The same average value of *V*_OC_ for both spray and spin SnO_2_ cells and slightly improvement of all the other electrical parameters of the solar device in sprayed cells is observed compared to spin-coated cells (see Figure S4). The slope of the *V*_OC_ and FF vs light intensity curves (Figure 5c,d) is smaller for devices based on spray SnO_2_ (1.32 *kT*/*q* and −4.2, respectively) compared to cells based on spin SnO_2_ (1.92 *kT*/*q* and −6.39, respectively), indicating high electron transporting in spray-based devices. The dark current density of both type of devices was likewise suppressed at negative bias (Figure 5e), suggesting the same hole-blocking capability for the two layers.

The operational stability was further evaluated by monitoring the effect of continuous 1 sun illumination at 55 °C on the PCE of unsealed spin and spray SnO_2_-based devices in ambient air under maximum power point tracking (MPPT, Figure 5f). Sprayed SnO_2_ layer slightly enhances the operational stability of the PSCs. Notably, the spray SnO_2_ cells maintained 80% of the initial PCE after 4 h of light soaking, whereas that of spin SnO_2_ reached the same loss after 2.5 h. One of the stability issues in PSCs originates from the electrons accumulated at the interface and the humidity permeation inside the device. Therefore, improving the connection ability of the ETL/perovskite interface can reduce the accumulation of charges and water/oxygen permeation at the interface, thus enhance the stability of the PSCs.^58,59^ Many research studies are recently focusing on the enhancement of operational stability of organic–inorganic PSCs by eliminating the charge accumulation at the ETL/perovskite interface.

**Figure 6:**
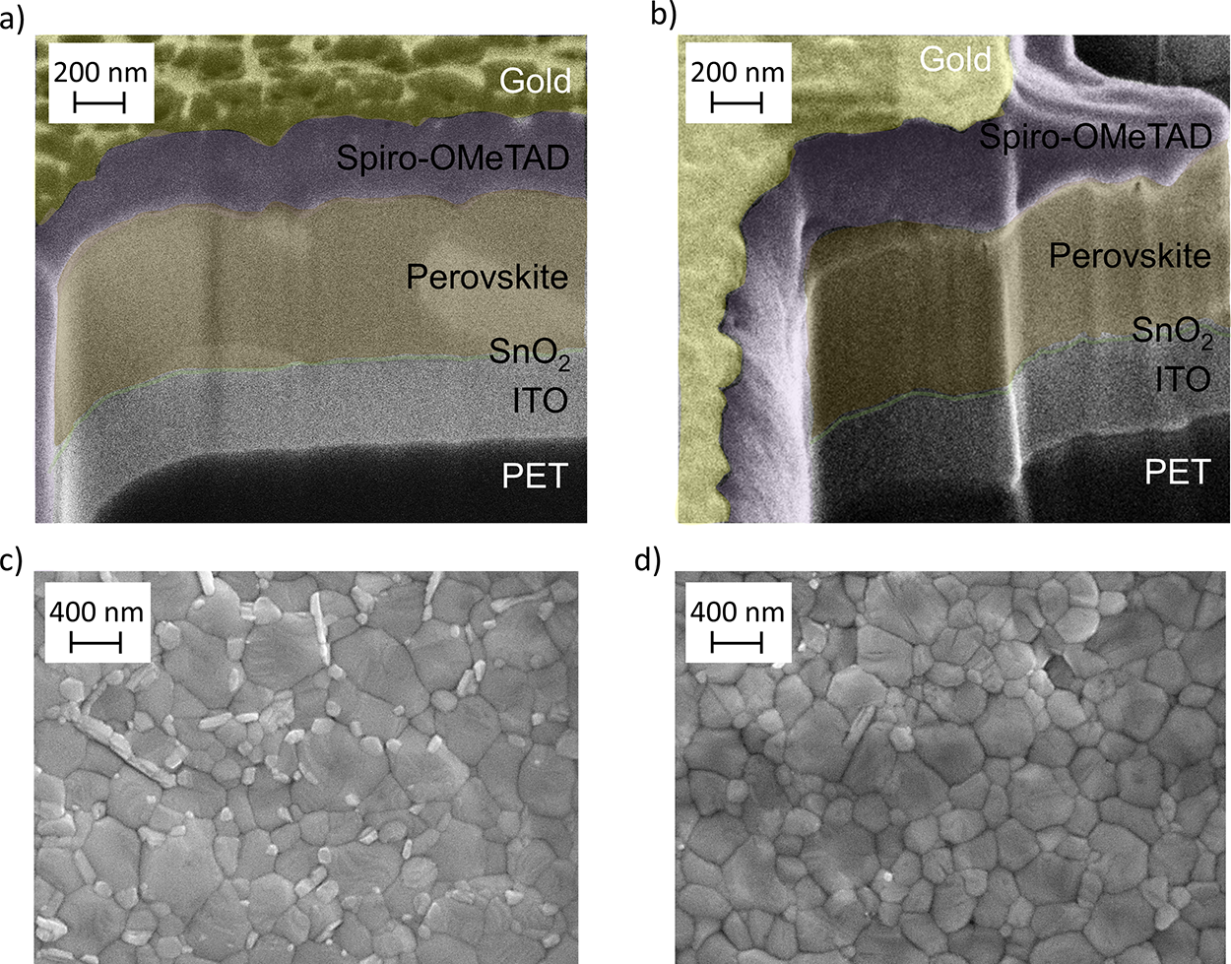
FIB-cut cross-sectional SEM images of a perovskite solar device based on (a) spin-coated SnO_2_ and (b) spray-coated SnO_2_. Top view SEM images of the perovskite layer grown on (c) spin-coated SnO_2_ and (d) spray-coated SnO_2_ films.

It is important to remark that not only the optical and electrical properties are important in defining the quality of the ETL in perovskite solar cells but also the ETL influence on the morphology of the perovskite film, which is grown on the top of the SnO_2_ layer. The vast literature has been devoted to the understanding of the effect of the perovskite film quality on the performances on solar cells, with the morphology being one of the most impacting properties.^35,60−62^ Therefore, we conducted SEM investigation to verify if the perovskite crystallization is affected by the SnO_2_ deposition technique. The SEM images of the perovskite layer on spray-coated and spin-coated SnO_2_ films are shown in Figure 6. In both cases, a dense packing of spherical grains with an equivalent diameter above 100–150 nm is evident, demonstrating that the deposition technique of SnO_2_ is minimally affecting the perovskite crystallization. However, the surface morphology of the perovskite crystals grown over the spin-coated layer showed the presence of small white grains, which may be excess of PbI_2_ crystals on the perovskite film surface.^63^ On the other hand, perovskite grown on sprayed SnO_2_ appeared as a much clear surface, without white grains and with slightly smaller perovskite grains. The increase in hysteresis in spin-coated SnO_2_ cells can also be related to the excess of PbI_2_,^17^ which consequently creates trap states for separated charges. In general, unfavorable high trap carrier and defect densities^64^ cause charge accumulation within the perovskite and at the adjacent interfaces.^65^ Since the less charge transport of the electron collection layer of spin-coated SnO_2_-based device compared to spray-coated one is demonstrated by the slope of the *V*_OC_ and FF vs light intensity curves (Figure 5c,d),^66^ charge accumulation mainly generates at the electron transport layer/perovskite interface.^65,67^ The accumulated charge at the ETL/perovskite interface is known to create a potential barrier and hence weaker electron transport, which might increase the risk of nonradiative charge recombination^65^ and most importantly, the light soaking causes severe device degradation when charges are accumulated at the interface.^68^ Stability improvement in FPSCs with a spray-coated SnO_2_ layer may be associated to the suppression of charge accumulation that occurs during the light soaking aging process in the PSCs (see Figure 5f).

This confirms that the spray technique for ETL deposition is an effective alternative to spin coating.

#### 2.2.2 Optimized Laser Scribe Process on Spray-Coated SnO_2_-Based FPSMs

**Figure 7:**
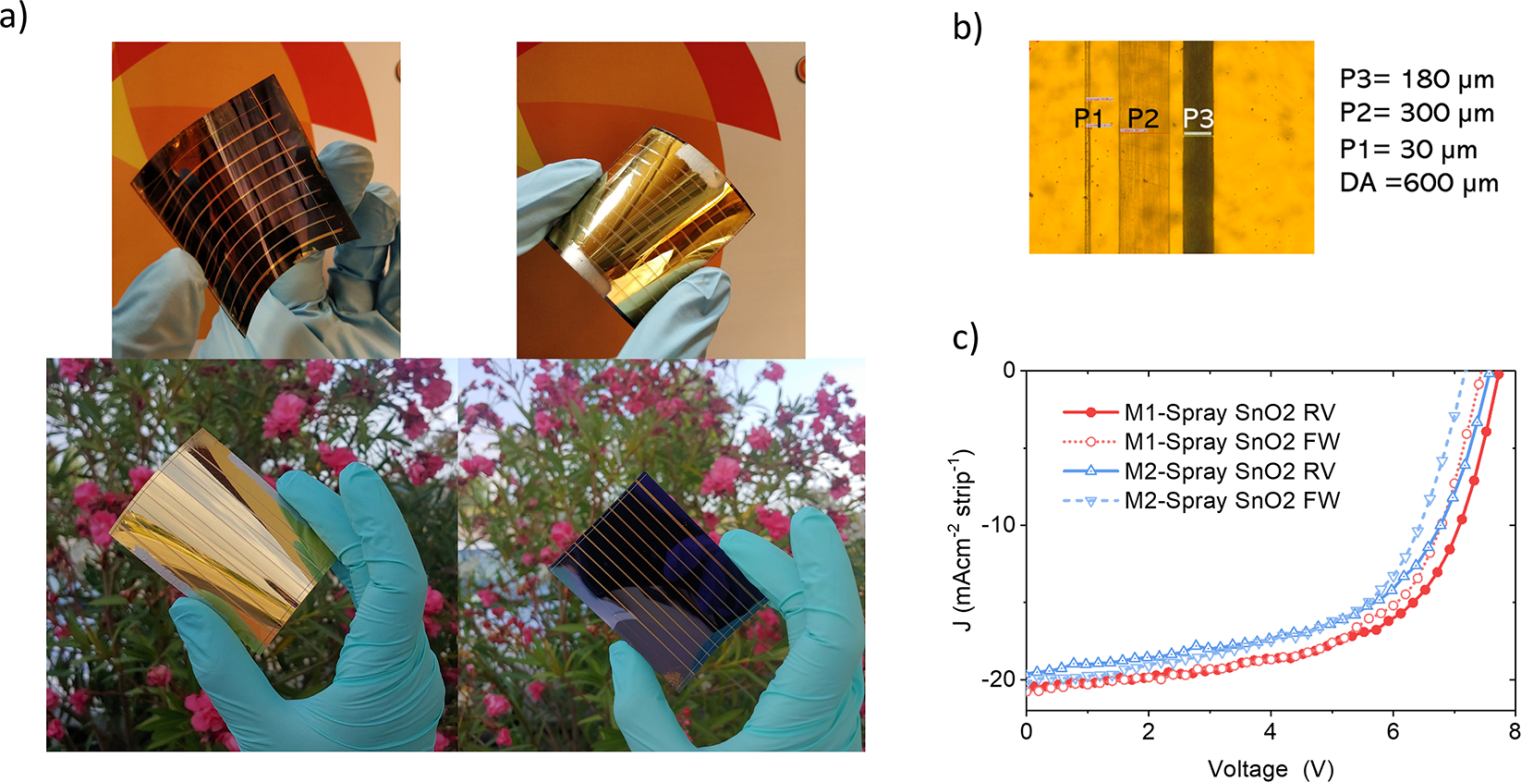
(a) Photograph of front and back of FPSM; (b) microscope image of the dead area; (c) *J*–*V* curves of FPSM based on spray-coated SnO_2_ films.

To investigate the effect of a larger active area on the module performance, two module layouts (Figure S2 and Figure 7) were designed by increasing both the number of cells and the cell length while keeping the cell width constant and equal to 4.5 mm, resulting in active areas of about 17 and 22 cm^2^, respectively. P1 and P3 were designed to be 30 and 150 μm, respectively. P2 width was fixed to 300 μm to guarantee the necessary contact area between evaporated gold (top electrode) and ITO (bottom electrode), even though it limited the module aperture ratio (AR), i.e., the ratio between the active area (AA) and the aperture area (AA + DA), which is approximately 87%, with the dead area (DA) being 600 μm overall (see Figure 7b).

Automated spray-coating was employed for the deposition of the SnO_2_ ETL as well as the optimized laser setting parameters for P2 scribing (i.e., 43 mW of laser power pulse, 100 mm/s of ablation scanning speed, and 15 μm of RSD), that improved the FF of the flexible mini modules up to 64%.

As reported in Table 2 and Figure 7c, the M1-SnO_2_ spray module with an active area of 16.84 cm^2^ achieved 12% PCE with FF of 60.8%, 7.72 V of *V*_OC_, and *J*_SC_ of 2.55 mA/cm^2^; the M2-SnO_2_ spray module with an active area of 21.84 cm^2^ yield 10.7% PCE with FF of 57.7%, 7.57 V of *V*_OC_, and *J*_SC_ of 2.45 mA/cm^2^. These results demonstrate that the FPSCs can be easily scaled-up when homogeneous layers can be deposited.

**Table 2:** Photovoltaic Parameters of Flexible Modules Based on Spray-Coated SnO_2_ Electron Transport Layers and Processed by Optimized P2 Laser Scribing (Laser Power = 43 mW, RSD = 15 μm, and 100 mm/s of Ablation Scanning Speed), and the Average of PCE Was Obtained from Five Modules

flex module	PCE (avg) (%)	*V*_oc_ (V)	*J*_sc_ (mA/cm^2^ strip^–1^)^a^	FF (%)	*J*_sc_ (mA/cm^2^)	active area (cm^2^)
M1-SnO_2_ spray rev scan	12.0 (10.5)	7.72	20.4	60.8	2.55	16.84
M1-SnO_2_ spray for scan	11.4 (9.9)	7.53	20.9	57.8	2.61	
M2-SnO_2_ spray rev scan	10.70 (9.86)	7.57	19.60	57.7	2.45	21.84
M2-SnO_2_ spray for scan	10.49 (9.26)	7.26	20.22	57.1	2.53	

a Current density (*J*_SC_) of a single strip.

When the device dimensions increase from the small cell size to the module size, the resulting performance loss is mainly caused by the reduced FF and *V*_OC_, usually the resistance of the conducting substrate or the back contact cause ohmic loss, consequently reducing FF and *V*_OC_.^48,69^

In our case, when the active area increased from 0.1 to 16.84 and 21.84 cm^2^, the PCE decreased by 21.5 and 30%, respectively (from 15.3% PCE to 12 and 10.7%), which is lower than the PCE drop suffered by PSCs in other upscaling works reported in the literature,^16,18,19^ i.e., greater than 25%, when the active area is increased from area 0.1 to 10 cm^2^.

**Figure 8:**
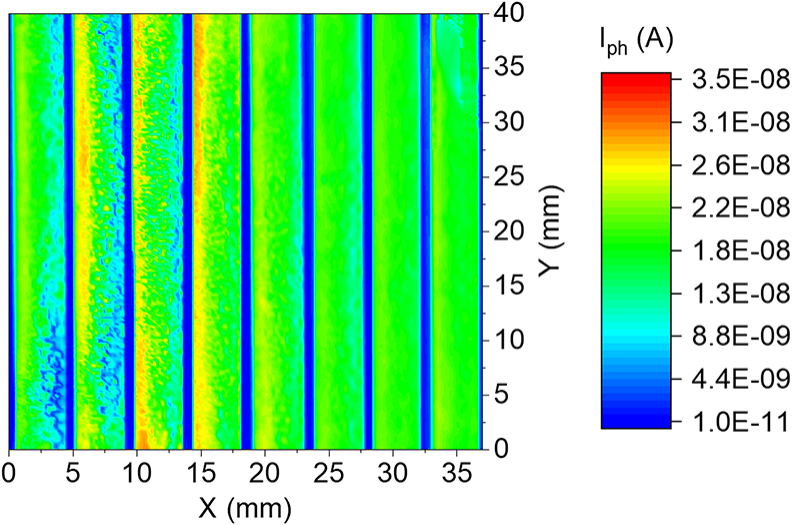
Light beam-induced current (*I*_ph_) map of flexible modules based on a sprayed SnO_2_ ETL, measured at a beam wavelength of 530 nm.

In order to assess the uniformity of large area perovskite modules, light beam-induced current (LBIC) measurements were performed by locally illuminating the module at the wavelength of 530 nm. LBIC maps can provide a precise spatial distribution of the current generation across the whole module.^24^ The LBIC map, displayed in Figure 8, shows that each cell of the module is efficiently separated from the adjacent one, as the dead areas of the module (dark blue) show 3 orders of magnitude lower currents than the active areas (highlighted in green to orange). We can therefore expect no significant interconnection losses, as demonstrated by the good fill factors of the fabricated modules, and a high *V*_OC_, resulting from the sum of the photovoltages of the 8-series-connected cells. Current generation is homogeneous over the whole active area of the module; small variation in photoresponse in areas close to the scribes can be attributed to a locally compromised wettability of the flexible substrate near P1 laser scribe lines, resulting from the different hydrophobicities of the ITO-ablated PET^70^ and PET/ITO.^57^ The irregularity of the left parts of the cells might be explained by the partial delamination of the gold used as the counter electrode and also to a slight tilt of the flexible module along that side, causing the module to be not as uniform as expected for the rest of the cells.

## 3 Conclusions

This work presents a thorough discussion and optimization of the laser process for flexible perovskite modules on large area by means of a UV raster scanning laser. Based on a systematic variation of the nano-pulse laser scribing parameters, on the morphological analysis of the scribed areas and on the electrical properties of flexible mini modules, the optimal process for successful laser-based series interconnection on flexible PET/ITO were identified.

Regarding the P2 laser scribing optimization, first, we analyzed the effect of different laser pulse powers and scanning speed on the quality of the scribes performed on a full device stack (without a top electrode). The optimal parameters ensuring efficient removal of overlying layers of the ITO were found to be laser pulse power equal to 43 and 46 mW with 100 mm/s of ablation scanning speed.

Then, we investigated the effect of the RSD on the performance of mini modules. At higher RSD, the optimum value results in an unfinished scribe width causing poor performance. For the RSD above the optimum value, a performance drop is caused by the high presence of cracks on the ITO layer. These observations are confirmed by the series resistance and fill factor derived from the *J*–*V* curve of devices.

Moreover, we successfully developed a simple and reproducible scalable deposition strategy using automated spray-coating to prepare uniform and dense SnO_2_ ETLs to obtain high efficiency, hysteresis-free, low-temperature fabricated planar PSCs on flexible PSCs as well as flexible PSCMs on plastics. In addition, the SnO_2_ spray-coating method has lower material consumption, larger producible area, and easier process compared to the spin-coating method. With this deposition technique, we achieved flexible PSC with a PCE of 15.3% (reverse scan) and 15% (forward scan) on 0.1 cm^2^ of active area.

The combined use of a UV nanosecond laser and large-area deposited SnO_2_ together with a proper optimization of the P2 scribing permitted to achieve flexible modules with 87% of aperture ratio, active area of 16.84 and 21.2 cm^2^, and a PC of 12 and 10.7%, respectively.

Due to the sturdiness and scalability of these techniques, this work opens an effective way to the fabrication of even larger area flexible PSM, in the view of a forthcoming industrialization of solution-process PV technology.

## 4 Experimental Section

### 4.1 Solar Cell Fabrication

After ultrasonic cleaning of the conductive PET/ITO substrate by IPA, an aqueous colloidal dispersion of SnO_2_ nanoparticles (15% in weight, Alfa Aesar) was spin-coated in air onto PET/ITO flexible substrates. For reference devices, spin coating was done at 6000 rpm for 45 s using 150 μL of SnO_2_-NP solution. For sprayed-SnO_2_, a diluted (7.5 wt %) dispersion of tin oxide nanoparticles in water was used. The precursor solution was placed in a reservoir within the automated spraying setup (Aurel), and it was continuously recirculated through a gear pump inside the spray nozzle. The solution was atomized at the tip of the nozzle into an aerosol by applying gas pressure using a compressed air. The flow rate, deposition velocity, and the path of spray and distance of the spray nozzle to the substrate were kept at 15 mL min^–1^, 500 mm/s linear path (with distance from lines of 7 mm), and 5.5 cm, respectively. The substrate temperature was set at 25 °C and air pressure at 1.5 bar. SnO_2_ solution was deposited at 60 μm of the aperture of the nozzle by fixing the spraying cycle to one time. SnO_2_ films were annealed in air at 100 °C for 40 min and then UV treated for 15 min before the perovskite deposition. The triple cation Cs_0.08_FA_0.78_MA_0.16_Pb(I_0.84_Br_0.16_)_3_ perovskite precursor solution was prepared by dissolving 547.4 mg mL^–1^ PbI_2_ (TCI), 87.1 mg mL^–1^ PbBr_2_ (TCI), 21.6 mg mL^–1^ MABr (Greatcellsolar), 166 mg mL^–1^ FAI (Greatcellsolar), and 19.4 mg mL^–1^ CsI in mixed *N*,*N*-dimethyl sulfoxide (DMSO) and *N*,*N*-diethyl formamide (DMF) solvents (1:3.16 by volume) by stirring for 24 h at room temperature. The as-prepared precursor solution was then spin coated in a glovebox (GB) onto the tin oxide films with two steps spinning, first, 1000 rpm for 10 s and then 5000 rpm for 30 s, 7 s before the end of the second spin coating step, and 150 μL of chlorobenzene was dropped on the substrates. Afterward, the perovskite layer was treated at 100 °C for 50 min in a nitrogen ambient. The hole transporting material (HTM), a 73.5 mg mL^–1^ solution of spiro-OMeTAD (Borun) in chlorobenzene, with the addition of 16.6 μL mL^–1^ lithium bis-(trifluoromethyl sulphonyl)imide (520 mg mL^–1^ in acetonitrile), 7.2 μL mL^–1^ cobalt(III) tris(bis(trifluoromethylsulfonyl)imide) (FK209, 0.25 M in acetonitrile), and 27 μL mL^–1^ 4-*tert*-butylpyridine, was spin-coated at 2000 rpm for 20 s in air. Finally, the cells were completed by thermal evaporation of 80 nm of Au as the top electrode.

### 4.2 Mini Module and Module Fabrication

PET/ITO substrates were etched with a UV Nd:YVO4 laser beam λ = 355 nm raster scanning laser with a repetition rate (rr) = 80 kHz and a pulse length = 15 ns to obtain the layout of the mini or large modules (P1 ablation). A 30 μm scribe was obtained with a laser power of 100 mW and a single-laser pass. The measured resistance between neighboring cells was higher than 200 MΩ, thus providing adequate electrical insulation.

For all module designs (Figure S2), the layout was based on series-connected cells, 4.5 mm wide. Mini modules were made of 3-series-connected cells with an overall active area of 2.34 cm^2^. Large modules included eight cells, with an overall active area of 16.84 and 21.84 cm^2^. The module aperture ratio (AR), i.e., the ratio between the active area and the aperture area (active + dead area), is approximately 87% (see Figure 7b).

After the P1 laser ablation, the patterned substrates were cleaned in an ultrasonic bath, using detergent with de-ionized water and isopropanol (10 min for each step). The etched flexible substrates were used for the SnO_2_ spray deposition and annealed the same as the small cells. The perovskite precursor solution and the spiro-OMeTAD solution were the same as for small cells. The amount of solution and anti-solvent (chlorobenzene) needed for the perovskite layer deposition for modules were 350 μL and 1 mL, respectively. Afterward, 300 μL of spiro-OMeTAD was used for HTL deposition. The spin-coating parameters for both perovskite and spiro-OMeTAD were the same as for the small cells.

P2 ablation was carried out to remove the SnO_2_/perovskite/spiro-OMeTAD stack on the interconnection areas, using the optimized laser parameters: laser pulse power equal to 43 mW, RSD of 15 μm, and with 100 mm/s of ablation scanning speed. Samples were then introduced into a high vacuum chamber (10^–6^ mbar) to thermally evaporate Au back contacts (nominal thickness of 100 nm).

Finally, P3 ablation was performed on the full stack using the same laser system (Nd:YVO4, λ = 355 nm). By using 74 mW of pulse power, 195 mm/s of ablation speed with 15 ns, an optimized P3 scribe with a width of 180 μm was obtained ensuring the electrical insulation between the counter electrodes of adjacent cells.

### 4.3 Characterization

The morphology of the perovskite layers and P2 laser-scribed strip processed were observed by scanning electron microscopy (SEM) FE-SEM ZEISS and confocal microscopy (Olympus Lext OLS 3100), respectively.

For the electrical characterization, we employed a custom-made system, which allows to simultaneously measure all the pixels of a device plate. They were carried out under a class A solar simulator (ABET Sun 2000) at 1000 W m^–2^ of flux density with an artificial solar spectrum of AM 1.5 G whose lamp was calibrated with a pyranometer. The current density vs voltage (*J*–*V*) characteristics of the perovskite solar devices were obtained from this system with a *J*–*V* scan rate of 33 mV/s. The FPSCs were masked with an aperture of 0.09 cm^2^ to define the active working area, and the FPSMs were measured by calculating the active area using a microscope.

The external quantum efficiency (EQE) spectra as a function of wavelength, *J*–*V* dark, light intensity-dependent *V*oc, FF, and MPPT light soaking stability of devices were collected using an optical power density-based measurement system (Arkeo–Cicci research s.r.l.). Absorbance of the perovskite film and ETL transmittance spectra were measured using a UV–vis spectrophotometer (Dymax EC-5000 lamp) in a wavelength range of 300–850 nm.

## Supporting Information Available

cross section SEM image of the perovskite cell; schematic representation of module layouts; transmittance of spray-coated SnO_2_ on 10 × 12.5 cm^2^ PET/ITO substrates; PV parameters of PSC cells endowed with sprayed and spin-coated SnO_2_ layers (PDF)
