# Supplementary material for: Laser-Scribing Optimization for Sprayed SnO2-Based Perovskite Solar Modules on Flexible Plastic Substrates
Source: ACS Appl Energy Mater. 2021 May 5;4(5):4507–18. doi: 10.1021/acsaem.1c00140 (PMC8288912; doi:10.1021/acsaem.1c00140)
Supplement: Supplementary file 1 — ae1c00140_si_001.pdf [file ae1c00140_si_001.pdf]

Supporting Information: Laser-scribing optimization for sprayed SnO_2-based perovskite solar modules on flexible plastic substrates

*Babak Taheri[1], Francesca De Rossi[1], Giulia Lucarelli[1], Luigi Angelo Castriotta[1], Aldo Di Carlo[1,2,3], Thomas M. Brown[1], Francesca Brunetti[1]**

[1] CHOSE, Department of Electronic Engineering, Università degli Studi di Roma Tor Vergata, Via del Politecnico 1, Rome, 00133, Italy

[2] LASE–Laboratory for Advanced Solar Energy, National University of Science and Technology MISiS, Moscow, Russia

[3] Institute for Structure of Matter, National Research Council (CNR-ISM), Rome, Italy

E-mail: francesca.brunetti@uniroma2.it

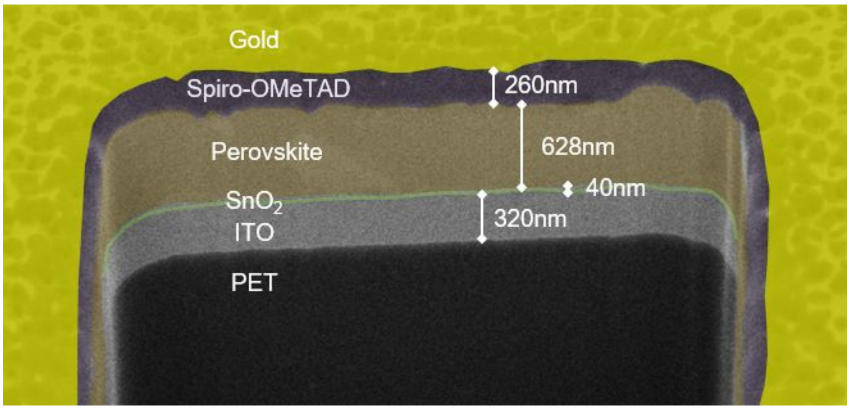

Figure S 1: FIB-cut cross-sectional SEM image of perovskite solar device.

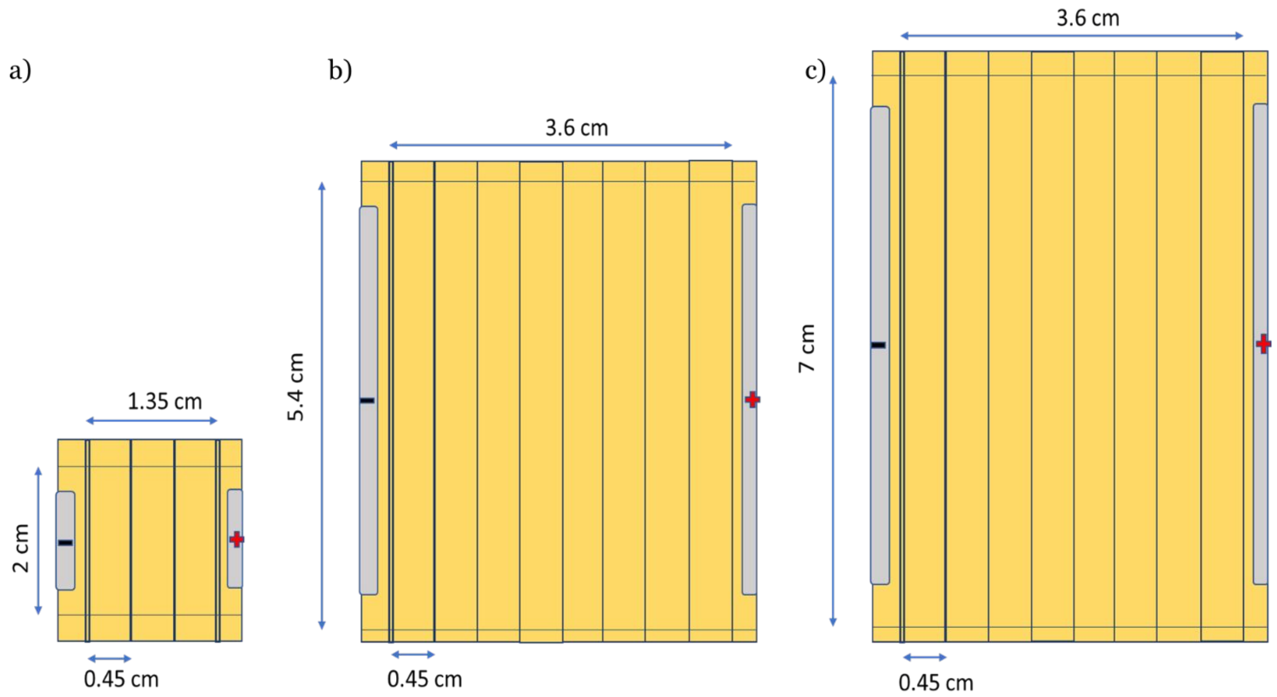

Figure S 2: Schematic representation of module layout a) mini modules with 2.34 cm^2 active area b) M1 modules with 16.84 cm^2 active area c) M2 modules with 21.84 cm^2 active area.

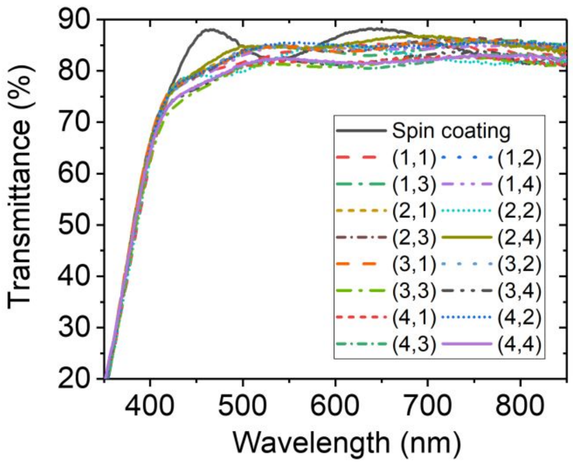

Figure S 3: Transmittance spectra of spray-coated SnO_2 samples from different portions of the 10×12.5 cm^2 flexible PET/ITO substrate used for the automatic spray coating process.

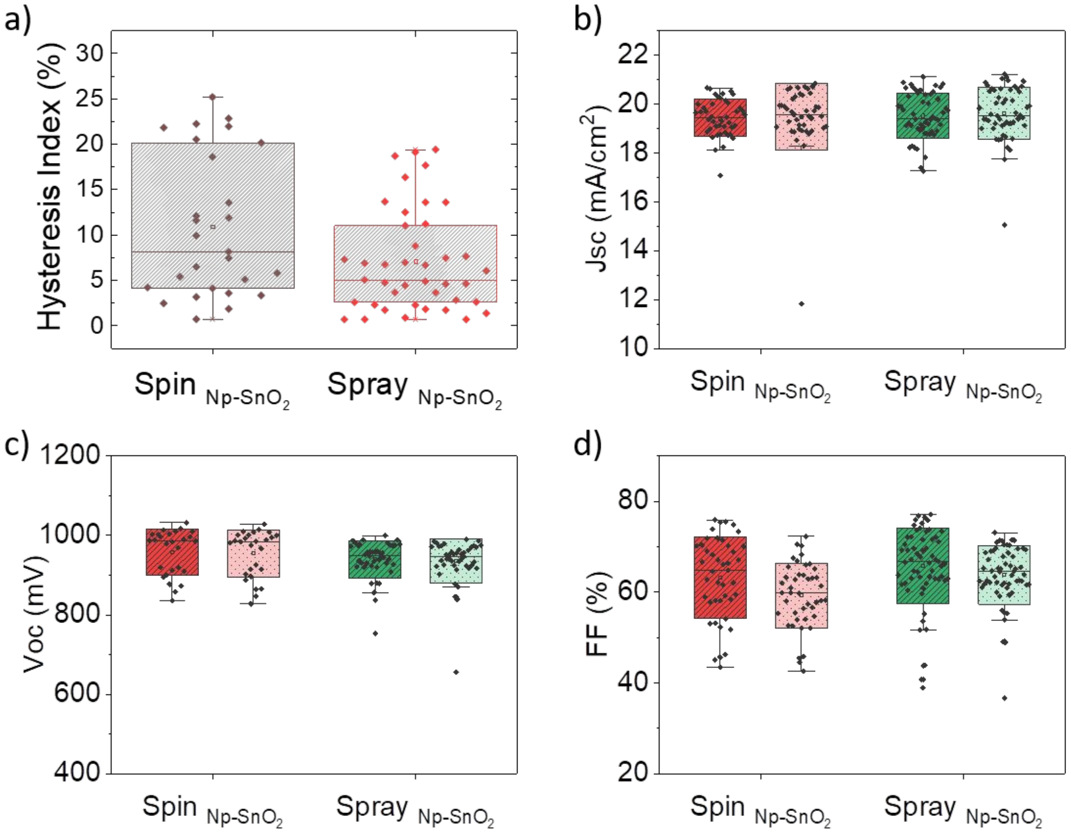

Figure S 4 : a) Hysteresis index calculated as follows: $(PCE_{rev} - PCE_{for}) / PCE_{rev}$; b) J_{SC}, c) V_{OC} and d) FF obtained from the J-V curves of FPSCs endowed with spray and spin-coated SnO_2 layers.
